# Comprehensive and Durable Modulation of Growth, Development, Lifespan and Fecundity in *Anopheles stephensi* Following Larval Treatment With the Stress Signaling Molecule and Novel Antimalarial Abscisic Acid

**DOI:** 10.3389/fmicb.2019.03024

**Published:** 2020-01-17

**Authors:** Dean M. Taylor, Cassandra L. Olds, Reagan S. Haney, Brandi K. Torrevillas, Shirley Luckhart

**Affiliations:** ^1^Department of Entomology, Plant Pathology and Nematology, University of Idaho, Moscow, ID, United States; ^2^Department of Biological Sciences, University of Idaho, Moscow, ID, United States

**Keywords:** *Anopheles*, malaria, abscisic acid, ABA, lifespan, fecundity, development

## Abstract

The larval environment of holometabolous insects determines many adult life history traits including, but not limited to, rate and success of development and adult lifespan and fecundity. The ancient stress signaling hormone abscisic acid (ABA), released by plants inundated with water and by leaf and root fragments in water, is likely ubiquitous in the mosquito larval environment and is well known for its wide ranging effects on invertebrate biology. Accordingly, ABA is a relevant stimulus and signal for mosquito development. In our studies, the addition of ABA at biologically relevant levels to larval rearing containers accelerated the time to pupation and increased death of *A. stephensi* pupae. We could not attribute these effects, however, to ABA-dependent changes in JH biosynthesis-associated gene expression, 20E titers or transcript patterns of *insulin-like peptide* genes. Adult females derived from ABA-treated larvae had reduced total protein content and significantly reduced post blood meal transcript expression of *vitellogenin*, effects that were consistent with variably reduced egg clutch sizes and oviposition success from the first through the third gonotrophic cycles. Adult female *A. stephensi* derived from ABA-treated larvae also exhibited reduced lifespans relative to controls. Collectively, these effects of ABA on *A. stephensi* life history traits are robust, durable and predictive of multiple impacts of an important malaria vector spreading to new malaria endemic regions.

## Introduction

In 2017, mosquito transmission resulted in 219 million new cases of malaria, 435,000 of which were fatal (World Health Organization [WHO], 2019). A large effort has been made to estimate the burden of malaria in endemic areas using defined parameters including abiotic factors, human clearance of malaria parasites, and mosquito life history traits, such as survival, population density, reproductive output, biting rate, and parasite development (Smith et al., 2018). Many important mosquito species contribute to parasite transmission, increasing the complexity of both surveillance and control efforts. Our focus is *Anopheles stephensi*, the Indian malaria mosquito, an aggressive malaria vector mosquito that has invaded and become established in Sri Lanka, Djibouti, and Ethiopia, with significant risk for range expansion into Somalia, Eritrea and Sudan (Faulde et al., 2014; Surendran et al., 2018; Seyfarth et al., 2019; Takken and Lindsay, 2019). In Djibouti, *A. stephensi* has been linked to a resurgence of severe infection with the human malaria parasite *Plasmodium falciparum* (Seyfarth et al., 2019), so increased focus on this species is relevant and timely for control.

Larval development in *A. stephensi* is rapid and, under favorable conditions, may be completed within a week from egg hatching. On the other hand, less than ideal larval environments, including for example over-crowding, have been shown to alter life history traits, significantly reducing parasite transmission (Moller-Jacobs et al., 2014; Murdock et al., 2017). Insect development is largely controlled by the regulatory effects of juvenile hormone (JH) and 20-hydroxyecdysone (20E) and our understanding of these details is derived primarily from studies in *Manduca sexta*, *Drosophila melanogaster* and *Aedes aegypti* (Jindra et al., 2013). In particular, JH acts to suppress differentiation of imaginal disk into adult structures when levels of 20E rise, instead maintaining larval structures and inducing larval-larval molts (Riddiford, 2012). In *M. sexta* and *D. melanogaster*, titers of JH decline to undetectable levels in the final larval instar due to reduced JH biosynthesis, elevated JH esterase activity and 20E-dependent transcription that induces metamorphosis (Vince and Gilbert, 1977; Brutis et al., 1990; Riddiford et al., 2000, 2010; Yin and Thummel, 2005; Liu et al., 2009). To date there has been no comprehensive analysis of JH titers over the course mosquito larval development. However, a significant body of work has been published on JH biosynthesis rates and transcriptional regulation of enzymes in adult *A. aegypti* (Noriega, 2014; Van Ekert et al., 2014). In this species, JH acid methyl transferase (JHAMT) is the ultimate enzyme in the principal JH biosynthesis pathway, converting inactive JH acid to active JH (Van Ekert et al., 2014).

Proteins and lipids stored from the larval diet contribute to proper ovarian development in female *A. aegypti*, with approximately half of the lipid stores carried forward to the adult stage (Zhou et al., 2004), such that females with lower larval protein reserves develop smaller follicles (Caroci et al., 2004). In addition to the direct effects of diet, nutrient status of newly emerged adults modulates the effects of insulin/insulin-like growth factor signaling (IIS) on JH synthesis, which remodels the fat body in preparation for a bloodmeal (Raikhel and Lea, 1983; Perez-Hedo et al., 2014). During the first gonotrophic cycle in *A. aegypti*, 80% of the lipids within the ovaries are derived solely from sugar meals acquired as adults (Ziegler and Ibrahim, 2001). Following the blood meal, amino acids derived from blood proteins activate the target of rapamycin (TOR) signaling pathway and ovary ecdysteroidogenic hormone is released from the brain, triggering 20E synthesis and release in the ovaries (Dhara et al., 2013). Following release from the ovaries, 20E signaling is initiated by 20E binding to the heterodimeric receptor comprised of the ecdysone receptor (EcR) and ultraspiracle (USP) to upregulate fat body synthesis of vitellogenin (Vg) that is transported to the developing eggs (Martìn et al., 2001). In the context of these changes, 20E delivered from the male during mating in *Anopheles gambiae* can also interact with the EcR, together with a novel protein Mating-Dependent Regulator of Oogenesis or MISO, to regulate oogenesis and the post-mating switch to monandry and oviposition (Baldini et al., 2013; Gabrieli et al., 2014). While this physiology is presumably conserved in *A. stephensi* based on the presence of an orthologous *miso* gene, both post-feeding and post-mating physiology likely also modulate the switch to physiological sensitivity to oviposition site attractants (Davis and Takahashi, 1980).

*Anopheles stephensi* females show a breeding habitat preference for natural bodies of water, ranging in size from small puddles to larger calm riverbeds (Manouchehri et al., 1976). Flood and ditch irrigation can impact the ecology of mosquitoes by creating new breeding sites and larval habitats, which are more attractive to gravid female mosquitoes than natural habitats (Mwangangi et al., 2010; Wondwosen et al., 2016). Initial flooding and fast water currents are destructive to larval survival, causing physical harm and reducing critical oxygen tension (Soleimani-Ahmadi et al., 2014). However, following flooding there is an increase in mosquito density and diversity, due to increased temporary breeding habitats (Rodrigures et al., 2017). During flooding, inundated plants can experience high levels of stress, which can result in the release of plant stress hormones into aquatic environments. For example, research has shown flooding of tomato plants increases the concentration of the stress hormone abscisic acid (ABA) in soil water by approximately 2.5-fold over control levels to ∼1.7 μM (Else et al., 1995).

Abscisic acid was first identified in plants, however, it is now recognized as a universal signaling molecule which acts as an effective regulator of stress responses and pathogen biology in plants, parasitic protozoa, sponges, hydroids, insects, and mammals (reviewed in Olds et al., 2018). The interaction of ABA and insects has been studied in several contexts. In the big-headed grasshopper, *Aulocara elliotti*, ingested ABA increased the number of eggs per female, however, eggs derived from ABA-treated females exhibited decreased viability (Visscher, 1980). Injection of ABA reduced protein uptake and Vg concentrations following consumption of a liver meal in the flesh fly *Sarcophaga bullata* (De Man et al., 1981). In addition, ABA injection appeared to act negatively on 20E signaling by delaying the peak of 20E by 16 h (De Man et al., 1981). ABA from nectar and pollen ingested by honeybees (*Apis mellifera*) can be detected in honey (Lipp, 1990) and ingestion of ABA by honeybee larvae can increase cold tolerance and cellular immunity (Negri et al., 2017; Ramirez et al., 2017).

In our previous studies, we observed that ingestion of an ABA-supplemented blood meal by female *A. stephensi* induced signaling kinases associated with a transient metabolic shift in the midgut, fueling immune-mediated killing of *P. falciparum* prior to completion of oocyst development (Glennon et al., 2016, 2017). Interestingly, ingested ABA did not decrease *A. stephensi* fecundity in the first gonotrophic cycle in contrast to our predictions based on the effects of ABA in *A. elliotti* and in *S. bullata* (Glennon et al., 2017). However, given the effects of ABA on metabolism and homeostasis of the *A. stephensi* midgut, on nutrient stores and 20E levels in other insects, and the potential that ABA in water used for oviposition could impact larval growth, we sought to understand whether ABA, at levels consistent with those released by inundated plants, could affect *A. stephensi* larval development, pupation and fitness of adult females emerging from treated larvae.

Our data show that, at concentrations in water as low as 1 μM, ABA accelerated *A. stephensi* larval development with varying effects on larval 20E levels and increased mosquito death at the time of pupation. Adult females derived from ABA-treated larvae exhibited significantly reduced fecundity over multiple gonotrophic cycles and significantly reduced lifespan, which was not altered by additional treatment of adult females with ABA. Accordingly, the effects of exposure of larval *A. stephensi* to ABA were both striking and durable, suggesting that manipulation of ABA levels in breeding sites, perhaps through nanoparticle release of this natural compound, could be used to reduce mosquito development and reproduction as well as adult survival that is required for completion of the extrinsic incubation period of malaria parasite development.

## Materials and Methods

### Mosquito Rearing

*Anopheles stephensi* Liston (Indian wild-type strain) were reared and maintained 27°C and 80% humidity with a 16/8-hour light/dark cycle. Adult mosquitoes were housed in 1 ft^3^ wire mesh cages and provided continuous access to 10% sucrose-soaked cotton pads. Three days after eclosion, adult female mosquitoes were allowed to feed on live mice sedated with ketamine (50 mg/kg) and xylazine (5 mg/kg) in sterile saline. All animal procedures were approved by the University of Idaho Animal Care and Use Committee. Mosquitoes were provided shallow cups of water to oviposit at 48 h after blood feeding. Eggs were gently washed into 5 L Nalgene pans with shallow water. Larvae were maintained in 5 L Nalgene pans on a solution of 2% powdered fish food (Sera Micron) and baker’s yeast in a 2:1 ratio for the first 3 days followed by Game Fish Chow pellet food (Purina) until pupation. Adult mosquitoes were collected for experiments within 12 h post-eclosion and housed in screened, 500 mL polypropylene Nalgene containers.

### Effects of ABA on *A. stephensi* Larval Development and Pupation

Larvae were collected at 36 h following transfer of eggs into Nalgene pans to reduce variability in the starting age among larvae used for these studies. For each treatment group, 100 larvae were placed in 500 mL polypropylene Nalgene containers with 200 mL water with or without 1, 10 or 100 μM ABA (Caisson Labs). Due to the light sensitivity of ABA, 50 mL of water from each container was removed daily and replaced with freshly made ABA-supplemented water to yield a final concentration of 1, 10 or 100 μM ABA. Low concentrations of ABA (1 and 10 μM) were based on published soil water concentrations of flooded tomatoes (Else et al., 1995) and on our data from submerged tomato leaves and roots in water (Supplementary Table S1); the highest concentration (100 μM) was based on ABA treatment of *A. elliotti* from previous studies (Visscher, 1980). Larvae were fed as above and maintained through pupation and eclosion to adults. After the first pupae were observed, pupae were collected every 12 h until no larvae remained. The numbers of pupae collected each day were recorded as “time to pupation” in days. Collected pupae were placed into cups with untreated water within cartons for adult eclosion; pupal survival was monitored until all adults had emerged and these data were recorded as the proportion of total pupae surviving through to adult eclosion. Based on this design, *A. stephensi* were exposed to ABA during the larval stage only. Five separate cohorts were used to complete biological replicates of this study.

### Effects of ABA on Adult *A. stephensi* Lifespan

Female mosquitoes derived from untreated, control larvae or from larvae treated with 1, 10 or 100 μM ABA were maintained in separate cartons with 10% sucrose-soaked cotton pads. At 3–5 days following eclosion, each group of mosquitoes was offered a “human blood meal” of washed human type O + erythrocytes (Interstate Blood Bank) suspended 1:1 (vol:vol) in heat-inactivated human type A + serum (Interstate Blood Bank). A similarly prepared blood meal was offered once weekly via a Hemotek feeder (Hemotek Ltd) until no mosquitoes remained alive. For one lifespan study, emerged adult female *A. stephensi* from each larval control and treatment group were split into two groups and treated as follows. One group of adults received an unsupplemented human blood meal each week whereas the other group was provided a weekly human blood meal supplemented with 100 nM ABA, which approximates the concentration of ABA present in blood in mice and humans with malaria (Glennon et al., 2016, 2018). Two days following blood feeding, females were given the opportunity to oviposit in a shallow water dish. Dead females were counted and removed from each group every 48 h. Two separate cohorts were used to complete biological replicates of the lifespan studies.

### Effects of ABA on *A. stephensi* Fecundity

At 3–5 days after adult eclosion from groups prepared as in section “Effects of ABA on Adult *A. stephensi* Lifespan,” female *A. stephensi* were allowed to feed on a human blood meal. Following feeding, engorged females were carefully removed and placed into individual 50 mL conical tubes with moist filter paper and allowed to oviposit. Following oviposition, the number of eggs were counted and the females were returned to their respective control or treatment cartons. All females were held until they were fed again the following week. This process of feeding followed by separation and oviposition was repeated until females were no longer receptive to blood feeding. Four separate cohorts of mosquitoes were used to complete biological replicates of these studies.

### qRT-PCR Assays for Relative Gene Expression

Relative transcript levels of *A. stephensi 3-hydroxy-3-methylglutaryl-coa reductase* (*hmg-r*), *juvenile hormone acid methyltransferase* (*jhamt*), *insulin-like peptides 1-5* (*ilp1-5*; Marquez et al., 2011), and *Vg* were determined by qRT-PCR (Supplementary Table S2). All data were normalized to transcript levels of *A. stephensi* housekeeping genes *ribosomal protein s7* (*rps7*) and *rps17*. For larval gene expression analyses, five larvae from control and ABA-treated water were collected and pooled for RNA isolation at 1, 2, 4 and 6 h following daily replacement of rearing water as described in section “Effects of ABA on *A. stephensi* Larval Development and Pupation.” For *Vg* expression analyses, five adult female *A. stephensi* were collected from control and ABA-treated larvae and pooled at 6, 12, 24, and 48 h post-blood feeding for RNA isolation. Adults were killed by briefly freezing at −20°C and pools of larvae or adults were placed in 500 μL Trizol (Invitrogen). RNA was extracted using the phenol-chloroform method according to manufacturer’s instructions. cDNA was synthesized using the QuantiTect reverse transcriptase kit (Qiagen) according to manufacturer’s instructions. cDNA concentrations were adjusted to 500 ng/μL with molecular grade water. Data were normalized to housekeeping genes and reported as Log_2_(2^–Δ^ ^Δ^ ^*Ct*^). For each treatment group 4-5 replicates were completed, each with three technical replicates.

### Effects of ABA on 20-Hydroxyecdysone (20E) Titer

20E titers were measured during larval and pupal development and in adult female *A. stephensi* derived from control and ABA-treated larvae. For these analyses, 40 larvae, 20 pupae or 20 adult female mosquitoes were collected and pooled for each time point from each group. Larval collections started at 3 days post-hatching and continued until pupae were detected at ∼7 days post-hatching. Larvae were collected once a day at 8 h following daily replacement of rearing water as described in section “Effects of ABA on *A. stephensi* Larval Development and Pupation.” The pupal stage of *A. stephensi* lasts ∼36 h; pupae were collected every 8 h for the duration of this stage. Adult female mosquitoes were collected within the first 8 h following eclosion. Samples were prepared for analysis by adding 500 μL of 100% chilled methanol to pooled insects, then sonicating on ice (Fisher Scientific Model 100) at level 4 for 5 s intervals. Samples were centrifuged at 5000 × *g* for 5 min and the resulting supernatant transferred to a new tube. Methanol extraction was performed a second time and supernatants were pooled. Pooled supernatants were dried under N_2_ stream and stored at −30°C until the 20E titers were measured using 20-hydroxyecdysone EIA kit (Arbor Assays), following manufacturer’s instructions. Three separate cohorts of mosquitoes were used to complete biological replicates of these studies.

### Effects of ABA on Adult Female *A. stephensi* Protein Content

To measure total protein content of adult female *A. stephensi* derived from control or ABA-treated larvae, 2 mosquitoes within 8 h of eclosion were homogenized in 10 mM dithiothreitol with 1 mM protease inhibitor cocktail (Sigma) in 100 μL loading buffer (Bio-Rad). Samples were boiled for 5 min and then centrifuged at 10,000 × *g* for 10 min at 4°C. Supernatants were transferred to new tubes and stored at −80°C. Total protein content was determined using Bradford reagent (Alfa Aesar) using bovine albumin serum (BSA) as a standard. Three separate cohorts of mosquitoes were used to complete biological replicates of these studies, each with three technical replicates.

### Statistical Analyses

All statistical analyses were performed using R statistical software version 3.5.3. Time to pupation and clutch sizes were analyzed by ANOVA and *post hoc* Tukey’s test. Proportions of *A. stephensi* laying eggs and dying as pupae were analyzed using likelihood ratio test of independence (GTest). 20E titers by day were analyzed by Student’s *t*-test. Data from qRT-PCR assays were normalized by 2^–ΔΔ*CT*^, log_2_ transformed and analyzed by ANOVA. Lifespan data were analyzed by two stage hazard rate analysis, in which the first stage is a log-rank test, and the second stage is used in cases where the hazard rates are not proportional and cross each other (Qiu and Sheng, 2008). Data across biological replicates within treatments were analyzed by ANOVA; if differences across replicates were not significant, replicate data were combined for analysis. Differences were considered significant at α ≤ 0.05. All figures were created using the ggplot2 package within R.

## Results

### ABA Treatment of *A. stephensi* Larvae Reduced Time to Pupation, but Not in Association With Rising 20E Titers

**FIGURE 1:**
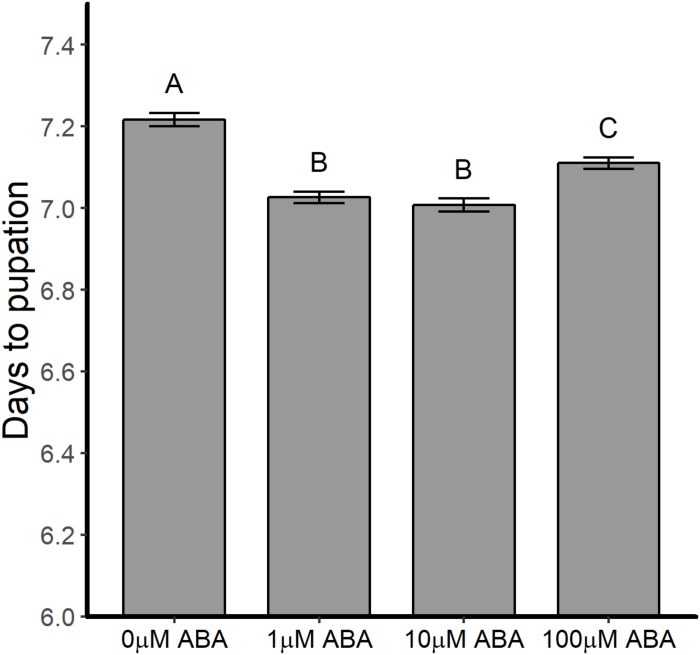
ABA treatment of larval *A. stephensi* reduced time to pupation. Larvae were exposed to 1, 10 or 100 μM ABA in rearing water as described in section “Effects of ABA on *A. stephensi* Larval Development and Pupation.” Time to pupation was reduced at all concentrations of ABA compared to control larvae (ANOVA *p* < 0.001). Data from five biological replicates were combined for analysis and are shown as mean ± SEM. Different capitalized letters indicate significant differences among controls and treatments. Control = 1456 larvae, 1 μM ABA = 1403 larvae, 10 μM ABA = 1382 larvae and 100 μM ABA = 1287 larvae.

Abscisic acid treatment of larval *A. stephensi* reduced mean time to pupation from 7.22 ± 0.77 days in untreated controls to 7.03 ± 0.67 days (1 μM ABA), 7.01 ± 0.76 days (10 μM ABA) and 7.11 ± 0.68 days (100 μM ABA) in treated larvae (Figure 1; ANOVA *p* < 0.001). Times to pupation in larvae treated with 1 and 10 μM ABA were not different (Tukey *p* = 0.877), but exhibited the shortest mean time to pupation relative to control. Larvae treated with 100 μM ABA had higher mean time to pupation relative to larvae treated with 1 μM ABA (Tukey *p* < 0.001) and 10 μM ABA (Tukey *p* < 0.001), but still pupated faster than untreated control larvae (Tukey *p* < 0.001). Although accelerated larval development would be expected to produce smaller adults (Lyimo et al., 1992), larval treatment with 1, 10, and 100 μM ABA was associated with increased size of emerged adult female *A. stephensi* relative to females derived from control untreated larvae (Supplementary Table S3).

**FIGURE 2:**
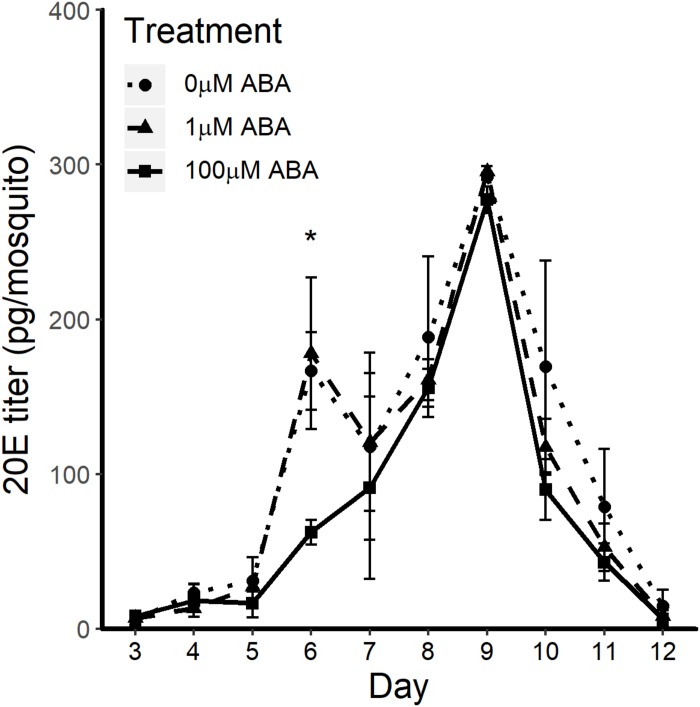
ABA treatment effects on 20E titers through larval development (days 1–7), during the pupal stage (days 8–9) and in newly emerged adult female *A. stephensi* (day 10) were minimal to undetectable. 20-hydroxyecdysone (20E) titers were measured from 20 to 40 individuals each day starting with second instar larvae from 3 days post-egg hatch through to 8 h post-eclosion of adult female mosquitoes. Larval exposure to 100 μM ABA reduced 20E titers on day 6 of larval development relative to controls (*t*-test *p* = 0.017), but no other differences were observed. Each biological sample was analyzed in triplicate to confirm assay reproducibility. Data from three biological replicates were combined for analysis and are shown as mean ± SEM.

In *D. melanogaster*, 20E regulates the timing of larval molts and, during the fourth and final instar, 20E titers rise to induce a cascade of transcriptional responses resulting in the physiological changes during the larval-pupal molt; studies in *A. aegypti* have shown similar 20E titers and expression of 20E-regulated genes (Margam et al., 2006; White and Ewer, 2014). Accordingly, we hypothesized that treatment of larvae with ABA might increase 20E titers earlier than in control larvae, resulting in reduced time to pupation in ABA-treated larvae. Based on the patterns observed in Figure 1, we analyzed 20E titers in larvae treated with the lowest (1 μM) and highest concentrations of ABA (100 μM) with untreated controls (Figure 2). Larvae exposed to 1 μM ABA showed no differences relative to controls in 20E titers over the course of larval development (days 1–7), during the pupal stage (days 8–9) or in newly emerged adult females (day 10). Larvae exposed to 100 μM ABA, however, had significantly reduced 20E titers on day 6 of larval development relative to controls (*t*-test *p* = 0.017), but no differences at any other timepoints. While there are no reports of 20E titers in *A. stephensi* larvae, our results are within the reported ranges for *A. aegypti* (Margam et al., 2006). These results suggested that the observed reduction in time to pupation in larvae exposed to ABA did not result from significantly elevated 20E titers in the final days of larval development.

### ABA Did Not Alter Transcript Expression of JH-Associated Genes in Larval *A. stephensi*

Since ABA treatment was not associated with increased 20E titers, we examined the expression of key genes in the mevalonate pathway and the branch pathway that synthesizes juvenile hormone (JH). Studies in *M. sexta* during larval-larval molts have demonstrated that JH titers remain elevated to suppress expression of 20E target genes and, in the final instar, JH titers decrease to undetectable levels, at which point 20E gene targets are upregulated and the pupal molt is initiated (Boulan et al., 2015). In the insect mevalonate pathway, 3-Hydroxy-3-Methylglutaryl-CoA Reductase (HMGCR) converts HMG-CoA to mevalonate, the precursor of farnesyl-pyrophosphate of the JH pathway. In the latter pathway, JH acid methyl transferase (JHAMT) methylates farnesoic acid to methylfarnesoate and is the terminal enzyme in JH biosynthesis in *A. aegypti* (Shinoda and Itoyama, 2003; Van Ekert et al., 2014). The gene expression profiles of *hmgcr* and *jhamt* were measured hourly for 6 h after daily replacement of rearing water as described in section “Effects of ABA on *A. stephensi* Larval Development and Pupation.” If expression levels of *hmgcr* and *jhamt* were down regulated in fourth instar larvae following exposure to ABA this could translate to reduced JH titers and earlier expression of 20E gene targets. However, we did not observe any significant changes in *hmgcr* expression relative to control in fourth instar larvae exposed to 1, 10 or with 100 μM ABA at 1 h (ANOVA *p* = 0.070), 2 h (ANOVA *p* = 0.275), 4 h (ANOVA *p* = 0.683) or 6 h post ABA treatment (ANOVA *p* = 0.239) relative to control (Supplementary Figure S1). There were also no significant changes in expression of *jhamt* in fourth instar larvae through the same 6 h period (ANOVA *p* = 0.144 for 1 h, *p* = 0.462 for 2 h, *p* = 0.054 for 4 h, *p* = 0.690 for 6 h post ABA treatment relative to control) (Supplementary Figure S1). Based on these data, it is unlikely that ABA treatment of *A. stephensi* fourth instar larvae alters JH titers in a pattern consistent with the effects of ABA on larval development.

### ABA Did Not Alter Expression of *ilp* Genes in *A. stephensi* Larvae

We previously demonstrated that ABA supplementation in a blood meal reduced the expression of *ilp3* and *ilp4* in adult female *A. stephensi* (Glennon et al., 2017), suggesting that ABA might also alter *ilp* expression in the larval stage. In *D. melanogaster* larvae, decreased drosulfakinin (DSK) has been associated with increased feeding and reduced food selectivity, with decreased *dsk* mRNA levels detected in *Dilp2,3,5* triple mutants but not *Dilp5* mutants (Söderberg et al., 2012). Based on these observations and because we saw no increase in 20E titers or changes in transcript expression of *hmgcr* or *jhamt*, we reasoned that ABA might decrease *ilp* expression in *A. stephensi* larvae in the fourth instar, which could increase food intake and trigger earlier pupation. Exposure of *A. stephensi* fourth instar larvae to ABA, however, through 6 h after daily replacement of rearing water as described in section “Effects of ABA on *A. stephensi* Larval Development and Pupation” had no effect on transcript expression of *ilp4* (ANOVA *p* = 0.362 for 1 h, *p* = 0.088 for 2 h, *p* = 0.276 for 4 h, *p* = 0.199 for 6 h post ABA treatment relative to control) or *ilp5* (ANOVA *p* = 0.733 for 1 h, *p* = 0.876 for 2 h, *p* = 0.692 for 4 h, *p* = 0.397 for 6 h post ABA treatment relative to control) (Supplementary Figure S2). We also examined expression of *ilp1-3* through the same 6 h period, but no significant differences relative to control were observed (not shown).

### ABA Increased the Percentage of *A. stephensi* Pupae Dying Prior to Adult Eclosion

**FIGURE 3:**
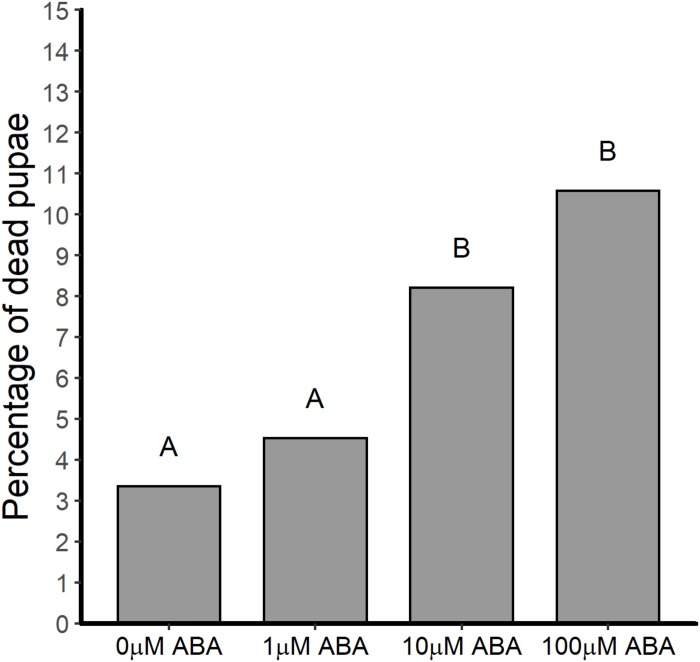
ABA treatment of larval *A. stephensi* increased the percentage of pupae dying prior to adult eclosion. While treatment of *A. stephensi* larvae with 1 μM ABA had no effect on pupae death relative to control (Gtest *p* = 0.249), larvae that were exposed to 10 and 100 μM ABA exhibited increased percentages of pupae death compared to control larvae (Gtest *p* < 0.001 and *p* < 0.001, respectively). Data are represented as percentages of pupae out of the total collected that died prior to adult eclosion. Data from three biological replicates were combined for analysis. Different capitalized letters indicate significant differences among controls and treatments. Control = 776 larvae, 1 μM ABA = 772 larvae, 10 μM ABA = 767 larvae and 100 μM = 700 larvae.

In addition to reducing the time to pupation, treatment of *A. stephensi* larvae with ABA resulted in a higher percentage of dead pupae (out of the total for each treatment group) relative to control. In the absence of ABA treatment, 3.4% of pupae died prior to adult eclosion (Figure 3). However, treatment with 10 and 100 μM ABA increased the percentage of dead pupae out of the total for each treatment group to 8.2 and 10.6% relative to control (Figure 3) (GTest *p* < 0.001 and *p* < 0.001, respectively). Accordingly, decreased time to pupation in ABA-treated larvae (Figure 1) was associated with increased pupal death prior to adult *A. stephensi* eclosion.

### ABA Reduced the Protein Content in Newly Eclosed Female *A. stephensi*

**FIGURE 4:**
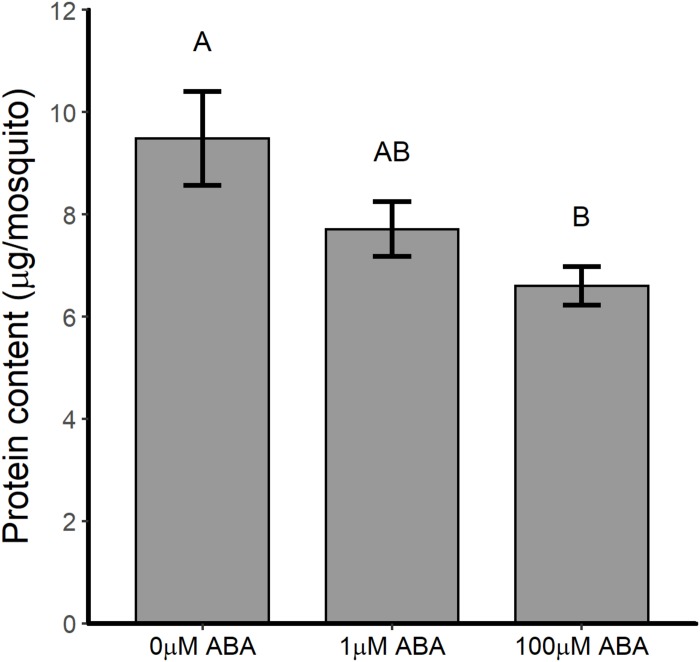
ABA treatment of larval *A. stephensi* reduced the protein content of newly eclosed adult females. Females emerged from larvae treated with 1 μM ABA exhibited a trend toward reduced protein content relative to controls (Tukey *p* = 0.214). Females emerged from larvae treated with 100 μM ABA exhibited reduced protein content relative to controls (Tukey *p* = 0.047), but were not significantly different from females emerged from larvae treated with 1 μM ABA (Tukey *p* = 0.493). Each biological sample was analyzed in triplicate to confirm assay reproducibility. Data from three biological replicates were combined for analysis and are shown as mean ± SEM. Different capitalized letters indicate significant differences among controls and treatments.

Female mosquitoes break down proteins derived from blood meals to amino acids, activating the TOR pathway, which signals fat body 20E synthesis and the upregulation of yolk protein precursor (YPP) genes in this tissue (Hansen et al., 2005). The nutrients stored during the larval stage are essential for JH-regulated fat body competency to respond to TOR activation such that malnourished mosquitoes exhibit reduced and delayed *Vg* transcript expression (Shiao et al., 2008). Based on these observations, we sought to examine the protein content of newly eclosed female *A. stephensi* to better understand the potential effects of ABA larval treatment on adult life history traits. Protein content of adult female *A. stephensi* derived from untreated control larvae was 9.5 ± 0.92 μg/mosquito (Figure 4). In comparison, protein content of adult females derived from larvae treated with 1 μM ABA was 7.7 ± 0.54 μg/mosquito trended downward relative to control but was not significantly different (Figure 4). However, protein content of females derived from larvae treated with 100 μM ABA, 6.6 ± 0.38 μg/mosquito, was significantly lower than that in controls, but not significantly different from that of females derived from larvae treated with 1 μM ABA (Figure 4). Accordingly, increased body size in adult females derived from larvae treated with 10 and 100 μM ABA (Supplementary Table S3) was associated with a trend toward decreased protein content (10 μM ABA) and significantly reduced protein content (100 μM ABA) relative to controls.

### Adult Female *A. stephensi* Derived From ABA-Treated Larvae Exhibited Reduced Fecundity

Given the reduction in protein content of newly eclosed female *A. stephensi* derived from ABA-treated larvae and the impact of nutritional status on JH-regulated fat body competency, we examined the fecundity of female *A. stephensi* derived from ABA-treated control larvae. In replicated assays, the percentages of females ovipositing and clutch sizes of female *A. stephensi* derived from ABA-treated larvae compared to females derived from untreated control larvae were variably reduced across three gonotrophic cycles.

**FIGURE 5:**
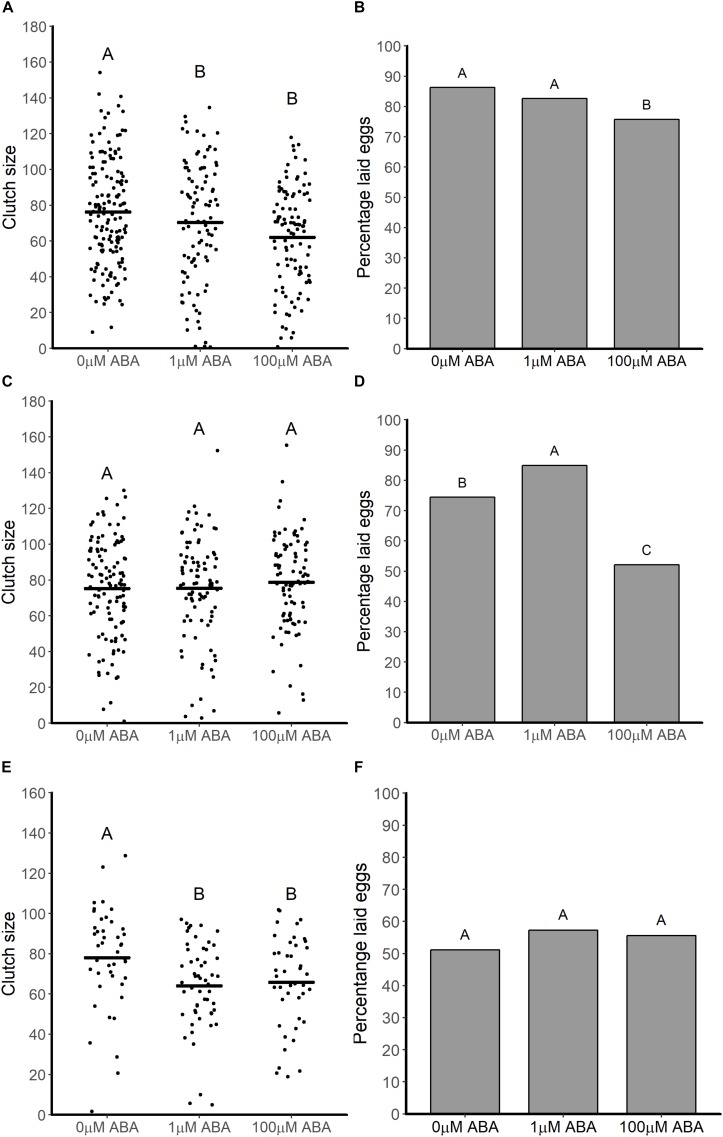
ABA treatment of larval *A. stephensi* variably reduced clutch sizes and percentages of ovipositing adult females across the first **(A,B)**, second **(C,D)** and third **(E,F)** gonotrophic cycles. **(A)** Female mosquitoes derived from larvae treated with 1 and 100 μM ABA had reduced clutch sizes in the first gonotrophic cycle compared to controls (Tukey *p* < 0.001 and *p* < 0.001, respectively). Each dot represents the clutch size of a single female mosquito from four biological replicates; black bars represent means. **(B)** Treatment of larvae with 100 μM ABA reduced the percentage of ovipositing females relative to controls in the first gonotrophic cycle (Gtest *p* = 0.013). Data are shown as the means from three biological replicates, *n* = 389. **(C)** Larval treatment with ABA had no effect on clutch sizes of adult female *A. stephensi* (ANOVA *F* = 0.009, *p* = 0.99) in the second gonotrophic cycle. Each data point represents the clutch size of a single female mosquito from four biological replicates; black bars represents means. **(D)** Treatment of larvae with 1 μM ABA increased the percentage of ovipositing females mosquitoes compared to controls in the (GTest *p* = 0.038), while treatment of larvae with 100 μM ABA reduced the percentage of ovipositing females compared to controls (GTest *p* < 0.001). Data are shown as the means of three biological replicates, *n* = 298. **(E)** Larval treatment with ABA reduced clutch size in the third gonotrophic cycle (ANOVA *p* = 0.012; Tukey *p* = 0.017 for 1 μM ABA, Tukey *p* = 0.034 for 100 μM ABA). Each dot represents the clutch size of a single female mosquito from four biological replicates, black bars represent the mean of each treatment. **(F)** Percentage of fully engorged *A. stephensi* that laid eggs was not affected by larval treatment with ABA (GTest *p* = 0.62). Data are shown as the means of three biological replicates, *n* = 141.

For the first gonotrophic cycle adult females derived from larvae treated with 1 and 100 μM ABA had reduced clutch sizes (ANOVA *F* = 15.195, *p* < 0.001; Tukey *p* < 0.001) relative to controls (Figure 5A). In addition to smaller clutch sizes in the first gonotrophic cycle, females derived from larvae treated with 100 μM ABA treatment were less likely to oviposit compared to controls (GTest *p* = 0.013) (Figure 5B). There was no effect of larval ABA treatment on clutch sizes in the second gonotrophic cycle (ANOVA *F* = 0.009, *p* = 0.99; Figure 5C) and an increase in the percentage of ovipositing females derived from larvae treated with 1 μM ABA mosquitoes compared to controls (GTest *p* = 0.038). As in the first gonotrophic cycle, however, there was a decrease in the percentage of ovipositing females derived from larvae treated with 100 μM ABA compared to controls (GTest *p* < 0.001; Figure 5D). By the third gonotrophic cycle, clutch sizes from females derived from larvae treated with ABA were again reduced relative to controls (ANOVA *F* = 4.49, *p* = 0.012; Tukey *p* = 0.017 for 1 μM ABA, Tukey *p* = 0.034 for 100 μM ABA; Figure 5E). However, in contrast to both prior gonotrophic cycles, there were no differences in the percentages of ovipositing females among the groups (GTest *p* = 0.62; Figure 5F).

### Post-blood Meal *Vg* Transcript Expression in Adult Female *A. stephensi* Derived From ABA-Treated Larvae Was Delayed Relative to Controls

**FIGURE 6:**
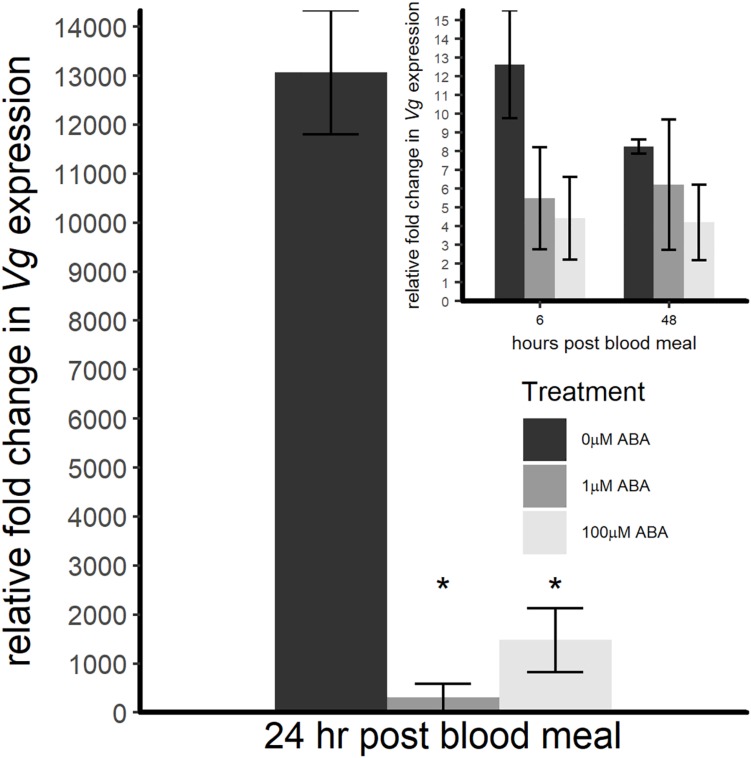
ABA treatment of larval *A. stephensi* reduced *Vg* transcript expression in adult female mosquitoes during the first 24 h following a blood meal. At 6 h post-blood meal (inset), there was no difference in *Vg* expression in females derived from larvae treated with 1 or 100 μM ABA compared to control (ANOVA *F* = 0.722, *p* = 0.519). At 24 h post-blood meal, however, *Vg* expression levels of females derived from larvae treated with 1 μM and 100 μM ABA were significantly reduced relative to controls (ANOVA *F* = 56.374, *p* < 0.001; Tukey ^∗^*p* < 0.001 for 1 μM ABA, Tukey ^∗^*p* < 0.001 for 100 μM ABA). At 48 h post-blood meal (inset), there was no difference in *Vg* expression in either ABA group compared to control (ANOVA *F* = 0.518, *p* = 0.616). Data are shown for three biological replicates as fold change normalized within treatment group to *Vg* expression before the blood meal.

Based on the effects of ABA larval treatment on adult fecundity, we sought to quantify the expression of *Vg*, the major YPP gene, in adult female *A. stephensi* within the first 48 h following a bloodmeal. The pattern of *Vg* expression in control females derived from untreated larvae followed the expected pattern of expression rising to a peak at 24 h, then declining (Figure 6). Adult females derived from larvae treated with 1 and 100 μM ABA showed significantly reduced *Vg* expression at 24 h post-blood meal (ANOVA *p* < 0.001, Tukey *p* < 0.001 for 1 μM ABA; Tukey *p* < 0.001 for 100 μM ABA) (Figure 6), suggesting that the effects of ABA larval treatment on adult female fecundity are at least partially explained by a significant reduction in *Vg* expression post-blood meal.

### Adult Female *A. stephensi* Derived From ABA-Treated Larvae Exhibited Reduced Lifespan

**FIGURE 7:**
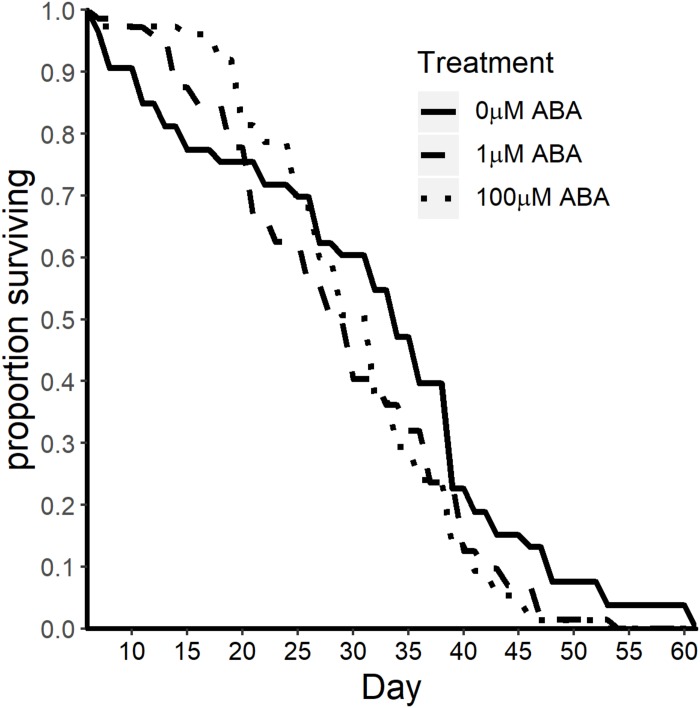
ABA treatment of larval *A. stephensi* reduced adult female survival. Survival curves of adult female mosquitoes derived from control untreated larvae and larvae treated with 1 μM ABA (log-rank *p* = 0.028) and 100 μM ABA (two-stage *p* = 0.025). Median survival was reduced from 34 ± 14.2 days in controls to 28 ± 10.78 days (1 μM ABA) and to 30 ± 14.22 days (100 μM ABA). Data are representative of two biological replicates.

Based on our observations of reduced protein content (Figure 4) and reduced fecundity (Figure 5) in adult female *A. stephensi* derived from ABA-treated larvae and evidence for tradeoffs between mosquito lifespan and reproduction (Harshman and Zera, 2007; Faiman et al., 2017), we predicted that ABA treatment of larvae might extend the lifespan of adult female *A. stephensi*. For this study, females derived from untreated control larvae and from larvae treated with 1 and 100 μM ABA received a blood meal once a week with maintenance on 10% sucrose between blood meals. For an additional study, adult females derived from control and treated larvae were each split into two groups, with one group receiving no ABA in the weekly blood meals and the other group receiving 100 nM ABA in the weekly blood meals. In contrast to our prediction, median survival in adult females derived from larvae treated with ABA was reduced to 28 ± 10.78 days (1 μM ABA; log rank *p* = 0.028) and to 30 ± 14.22 days (100 μM ABA; two-stage *p* = 0.025) relative to the untreated control median lifespan of 34 ± 14.2 days (Figure 7). Consistent with previous observations of no effect of ABA in blood on adult female lifespan (Glennon et al., 2017), the addition of 100 nM ABA in the blood meal had no effect on adult lifespan nor did it alter the effects of larval treatment with ABA on adult lifespan (Supplementary Table S4).

## Discussion

Taken together, our data demonstrate that the effects of treating *A. stephensi* larvae with ABA are durable, starting with accelerated pupation and increased pupal death and lasting through multiple gonotrophic cycles to reduce fecundity and adult female survivorship. These effects indicate the potential for multiple population level impacts on mosquito density, biting and pathogen transmission through combined reductions in immature stages, fecundity and lifespan at ABA concentrations that could occur under natural conditions (Else et al., 1995). In trying to understand these effects of ABA, we examined some obvious developmental cues in our studies. Accelerated pupation in holometabolous insects can result from blocking JH synthesis or activity. For example, removal of the corpora allata, which generates JH, results in precocious pupation in *Manduca sexta* due to a reduction in the critical weight threshold (Nijhout and Williams, 1974). Despite the obvious similarities in accelerated pupation, ABA had no effect on the expression of the JH synthesis-associated genes *hmgcr* and *jhamt*. Further, ABA affected 20E titers on only a single day of larval development (day 6) at the highest concentration of ABA (100 μM), affirming minimal to no effect of ABA on the interacting effects of JH and 20E. Our data contrast with the reported effects of ABA on 20E in adult *S. bullata* (De Man et al., 1981), suggesting the likely possibility that the effects of ABA vary to some degree across insect species. In *D. melanogaster*, reduced food seeking behavior is mediated by the overexpression and release of ILP2 and ILP4 (Wu et al., 2005), suggesting that reduced expression of *ilp*s in our ABA treated larvae might be associated with increased food seeking behavior. However, expression of *ilp* genes in fourth instar *A. stephensi* larvae was not altered by ABA treatment. While the current state of technology is not sufficient to quantify ILPs in *A. stephensi*, we can reasonably conclude that JH, 20E and changes in ILP levels are not mediating the acceleration of development by ABA. Future work regarding potential effects of ABA on *A. stephensi* larval feeding behavior could focus on sulfakinin and neuropeptide F, both of which modulate feeding behavior in *D. melanogaster* larvae (Söderberg et al., 2012; Fadda et al., 2019). Both neuropeptides are also known to be conserved in *A. gambiae* (Strand et al., 2016) and are represented as orthologs in *A. stephensi*^1^.

The protein content of newly eclosed adult females derived from larvae treated with ABA was reduced, perhaps as a consequence of accelerated development and increased adult size. Following a blood meal, malnourished mosquitoes have both delayed and reduced *Vg* transcript abundance and may require a second blood meal for proper egg development (Shiao et al., 2008). The reduction in *Vg* expression that we observed is consistent with reduced Vg levels in *S. bullata* injected with ABA (De Man et al., 1981) and reduced Vg levels reported for malnourished *A. aegypti* (Shiao et al., 2008). Reduced protein content has been shown to result in smaller follicle size and increased resorption of oocytes (Caroci et al., 2004; Clifton and Noriega, 2011). Our observed association between reduced *Vg* mRNA expression and decreased fecundity is consistent with other studies showing 50% of Vg depleted mosquitoes still produced mature eggs (Rono et al., 2010). In our studies, reduced protein content of newly eclosed adult females likely contributed to lower clutch sizes and reduced oviposition in the first gonotrophic cycle (following a blood meal at 4 days in week 1). Patterns of oviposition and fecundity in the second and third gonotrophic cycles (weeks 2–3) could reflect the fact that nutrients for optimizing reproduction and preserving the soma are limited. In fact, the inflection points in our lifespan data – which occur at 21 days (Figure 7) for 1 μM ABA and 26 days for 100 μM ABA – are consistent with the possibility that the cost of reproduction, even at reduced levels, outweighs any further investment in the soma of females derived from treated larvae relative to controls, which have consistently higher survivorship after these timepoints.

While dietary restriction and the resulting impacts on nutrient stores have been associated with lifespan extension in *Anopheles* mosquitoes (Faiman et al., 2017), we observed reduced survivorship with reduced body protein levels in *A. stephensi* derived from larvae treated with ABA. Reduced survival of ABA-treated mosquitoes was unexpected as weekly supplementation of adults with ABA in blood meals did not change adult female survival (Glennon et al., 2017). Our data indicate that the substantial effect of ABA on adult lifespan carries over from the larval stage and across not one, but two developmental transitions.

Study of the role of plants and plant biology in regulating mosquito life history has focused on the characteristics of oviposition sites that are shaped by both wild and cultivated plant species (Omlin et al., 2007; Overgaard, 2007; Eneh et al., 2016; Wondwosen et al., 2016; Asmare et al., 2017; Wondwosen et al., 2017; Wondwosen et al., 2018), on nectar feeding and its effects on mosquito physiology (Nikbakhtzadeh et al., 2014; Nyasembe et al., 2014; Jacob et al., 2018), the potential role of invasive plants in promoting malaria parasite transmission (Stone et al., 2018), and the utility of plant-derived compounds as novel insecticides (Govindarajan et al., 2008; Nathan et al., 2008; Elango et al., 2009; Elimam et al., 2009). Here, we have taken the relationship between plant biology and mosquito biology a step further, connecting the effects of ABA, a universal signaling molecule first described in and well known from plants (Olds et al., 2018), at concentrations detected in water with submerged plant tissue that can alter substantial features of mosquito growth, development and survivorship across immature and adult stages. Together with our previous studies on the effects of ABA on *P. falciparum* development in *A. stephensi* (Glennon et al., 2017), the association of elevated blood levels of ABA with asymptomatic malaria in humans and reduced infection and disease pathology in our animal model of malaria (Glennon et al., 2018), the effects of ABA on the life cycle of malaria are both comprehensive and complex and will become undoubtedly more so with continued studies of mechanisms underlying this biology.

## Data Availability Statement

The datasets generated for this study are available on request to the corresponding author.

## Ethics Statement

All animal procedures were approved by the University of Idaho Animal Care and Use Committee.

## Author Contributions

DT and SL designed the experiments and wrote the manuscript. RH designed and conducted the fecundity studies. BT assisted in completing various studies and provided input for experimental design. CO contributed to writing and editing the manuscript.

## Conflict of Interest

The authors declare that the research was conducted in the absence of any commercial or financial relationships that could be construed as a potential conflict of interest.

## Footnotes

1 www.vectorbase.org
