# Supplementary material for: Comprehensive and Durable Modulation of Growth, Development, Lifespan and Fecundity in Anopheles stephensi Following Larval Treatment With the Stress Signaling Molecule and Novel Antimalarial Abscisic Acid
Source: Front Microbiol. 2020 Jan 17;10:3024. doi: 10.3389/fmicb.2019.03024 (PMC6979008; doi:10.3389/fmicb.2019.03024)
Supplement: Supplementary file 1 [file Table_1.DOCX]

**Supplementary Material**

| Leaf | ABA concentration (µM) |
| --- | --- |
| 6 hours | 2.22 ± 0.46 |
| 24 hours | 1.39 ± 0.12 |
| 48 hours | 2.90 ± 0.66 |
| Root | **ABA concentration (µM)** |
| 6 hours | 1.03 ± 0.56 |
| 24 hours | 1.56 ± 0.30 |
| 48 hours | 0.51 ± 0.29 |
| Leaf + Root | **ABA concentration (µM)** |
| 6 hours | 0.65 ± 0.08 |
| 24 hours | 0.89 ± 0.15 |
| 48 hours | 1.48 ± 0.57 |

**Table S1.** Concentrations of ABA detected over time after submerging 2 g of *Solanum lycopersicum* (tomato) leaves, roots or leaves and roots in 200 mL of water. At 6, 24, and 48 hours, 1mL samples of water from each treatment were collected for analysis by LC-MS/MS. Samples were analyzed in triplicate, with an internal standard of deuterated 6-ABA. Data are shown as mean ± SEM.

| qRT-PCR primer sequences | | |  |
| --- | --- | --- | --- |
| Gene | **Forward** | **Reverse** | **Accession numbers** |
| *Vg* | 5’-CAACATCATGTCCAAGTCGGAGGTGA-3’ | 5’-CTTGAAGCTTTCGTGCTCTTCCTCCG-3’ | ASTEI03705 |
| *hmgcr* | 5’-GCGACACAACAGATCATCGGTAGC-3’ | 5’-AGATGATGGTGGATTCGTTTCACCAC-3’ | ASTEI00165 |
| *jhamt* | 5’-TCAGCACACTGCGTTCTCCAAC-3’ | 5’-GGGCGAACGAGACAGTTGGTT-3’ | ASTEI05976 |
| *ilp1* | 5’-GCTCGCTTCACTTGGTGTAACA-3’ | 5’-AAAGAAGCGAATGAAGTTTGATGA-3’ | ASTEI05349 |
| *ilp2* | 5’-TAACCGCCGATCGCTTCT-3’ | 5’-GCAGTGTGGCTACCTCAACCTT-3’ | ASTEI05346 |
| *ilp3* | 5’-GCGCGTCGATCTACAGTTTGA-3’ | 5’-GGTCGTGTCCGTCTTCATGA-3’ | ASTEI05348 |
| *ilp4* | 5’-GAGTTGACATCACAGTGGTTTAGGA-3’ | 5’-TCGCATCCGTGCCTTAATG-3’ | ASTEI05347 |
| *ilp5* | 5’-CAGTCGGCGGGACAAAAT-3’ | 5’-CGTAGGCCACTTCACGATCA-3’ | ASTEI00942 |
| *rps7* | 5’-GATTGCTGGTTTCGTGACCCATTTGA-3’ | 5’-GGATCCACCTCAATGATGTCCTGC-3’ | ASTEI05335 |
| *rps17* | 5’-GATTGCTGGTTTCGTGACCCATTTGA-3’ | 5’-GGATCCACCTCAATGATGTCCTGC-3’ | ASTEI10371 |

**Table S2.** Primer sequences and accession numbers for qRT-PCR assays

|  | Wing size (millimeters) | | | |  |
| --- | --- | --- | --- | --- | --- |
| Replicate | **Control** | **1 µM ABA** | **10 µM ABA** | **100 µM ABA** | **ANOVA p** |
| 1 | 3.04 ± 0.14 | 3.03 ± 0.11 | **3.12 ± 0.10** | **3.15 ± 0.12** | <0.001 |
| 2 | 3.15 ± 0.07 | 3.17 ± 0.10 | 3.17 ± 0.10 | 3.16 ± 0.10 | 0.737 |
| 3 | 3.12 ± 0.10 | **3.38 ± 0.17** | **3.36 ± 0.12** | **3.24 ± 0.11** | <0.001 |

**Table S3.** Mean wing size of adult female *A. stephensi* emerged from control untreated larvae or from larvae treated with 1, 10 or 100 µM ABA. In two of three replicates, larval treatment with 10 and 100 µM ABA was associated with increased wing size of emerged female mosquitoes relative to controls. In a single replicate, 1 µM ABA larval treatment was associated with increased wing size of newly eclosed female mosquitoes compared to controls. There was no effect of larval treatment with 1 µM ABA in any replicate. Bold, underlined text indicates significance (p < 0.05) relative to control mosquitoes.


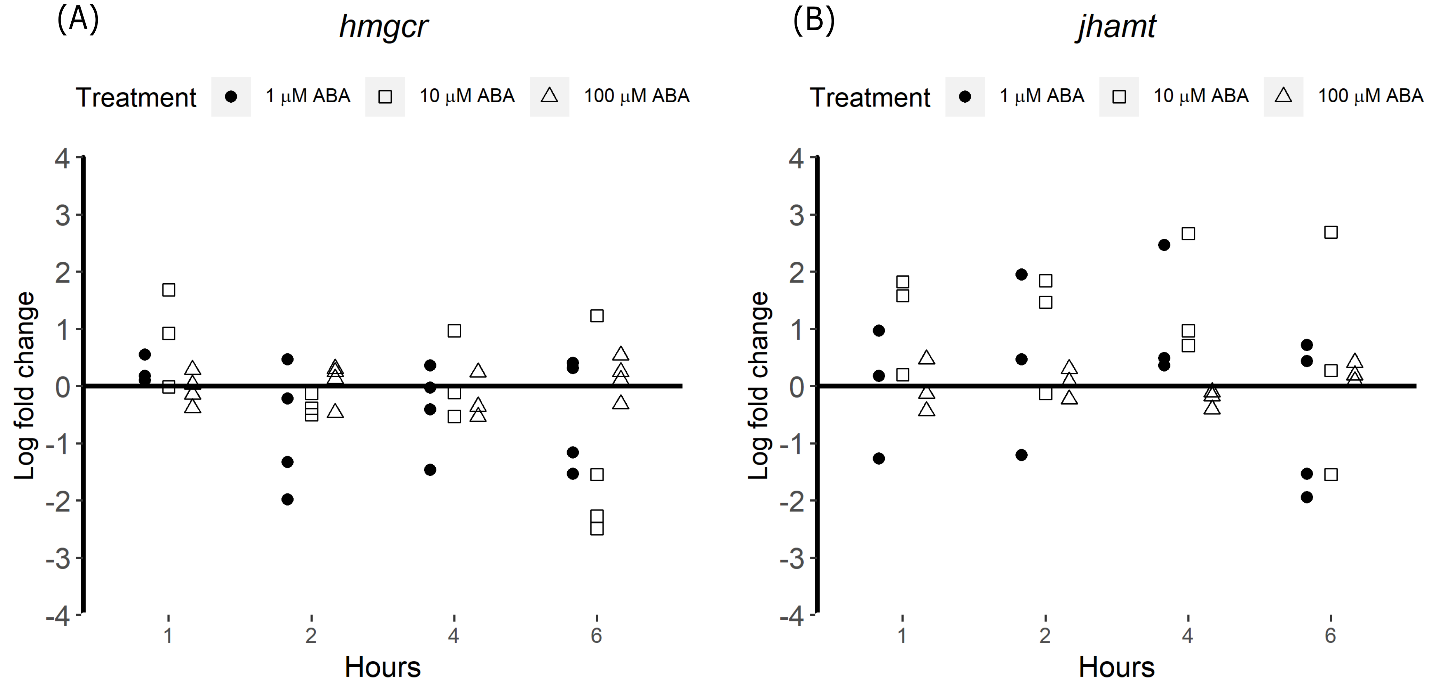


**Figure S1.** ABA treatment had no effects on transcript expression of *3-hydroxy-3-methylglutaryl-coa reductase* (*hmgcr*; **A**) and *juvenile hormone acid methyltransferase* (*jhamt*; **B**) in 4^th^ instar *A. stephensi* larvae relative to controls through 6 hr following daily replacement of rearing water as described in Methods 2.2. Each data point represents a pool of five 4^th^ instar mosquito larvae that were collected from five separate cohorts of *A. stephensi*. Three technical replicates were performed with each biological replicate sample to ensure assay reproducibility. Data are shown as Log_2_-fold change expression over control, which is represented as the black line at “0”.


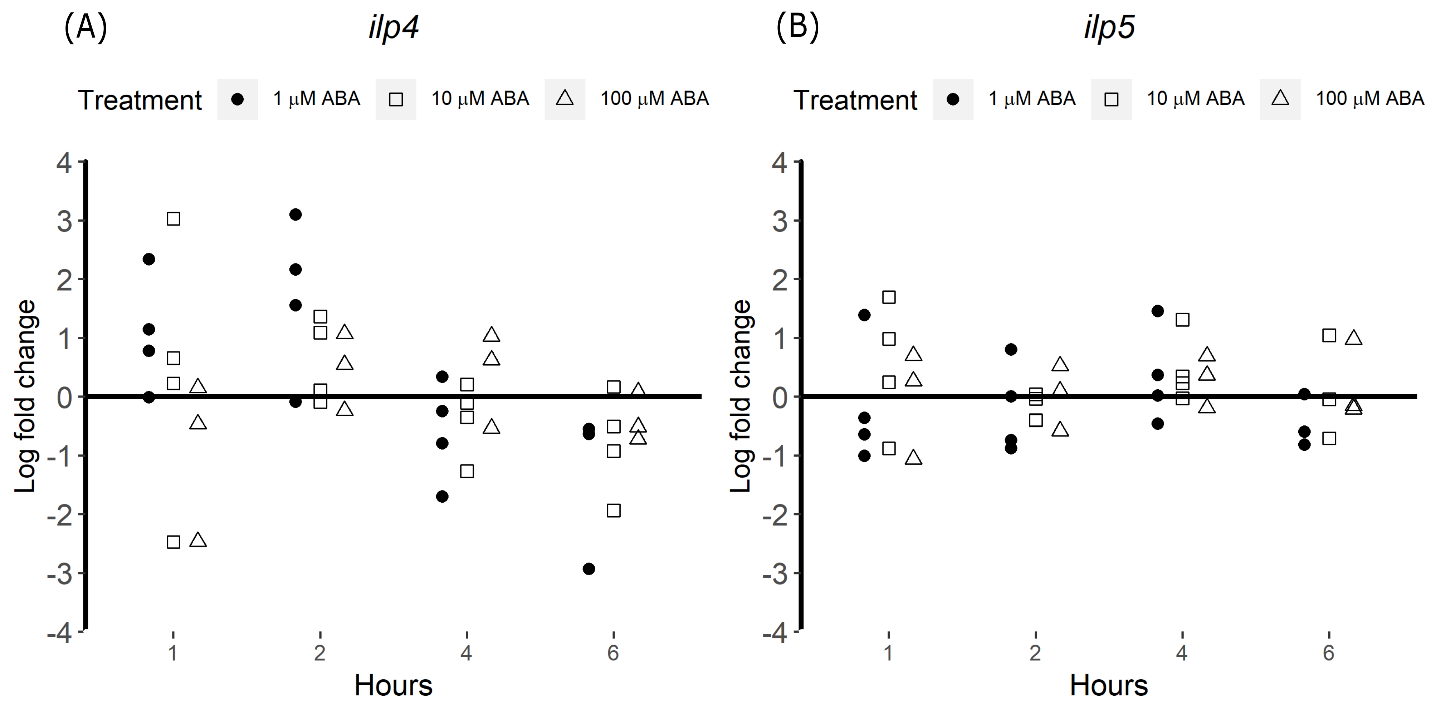


**Figure S2.** ABA treatment had no effects on transcript expression of *ilp4* (A) and *ilp5* (B) in 4^th^ instar *A. stephensi* larvae relative to controls through 6 hr following daily replacement of rearing water as described in Methods 2.2. Each data point represents a pool of five 4^th^ instar mosquito larvae that were collected from five separate cohorts of *A. stephensi*. Three technical replicates were performed with each biological replicate sample to ensure assay reproducibility. Data are shown as Log_2_-fold change expression over control, which is represented as the black line at “0”.

**
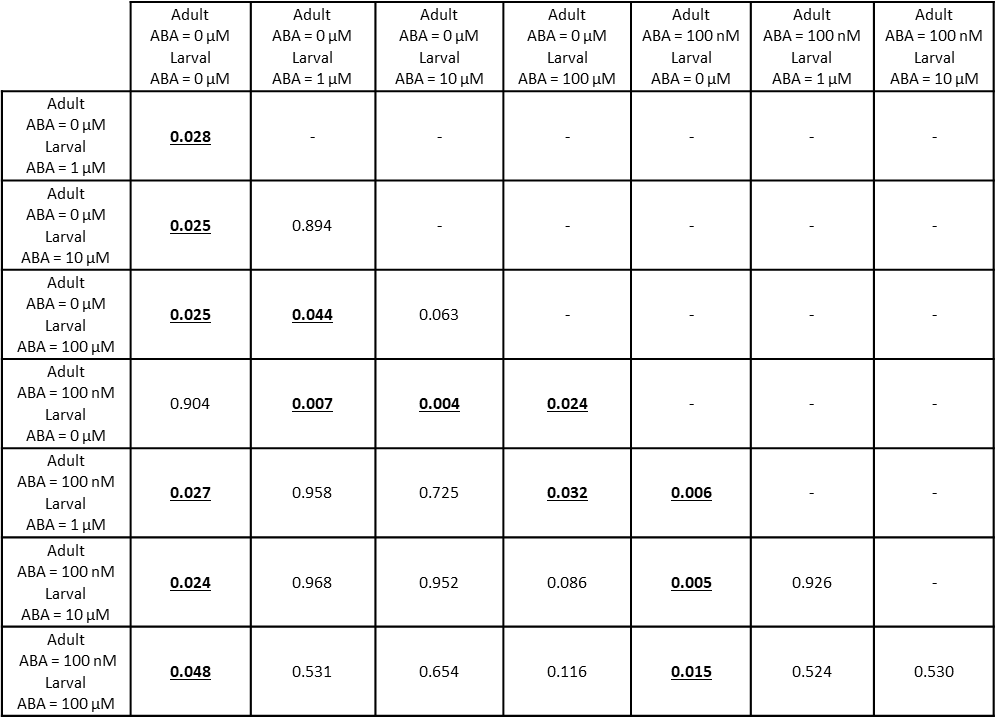
**

**Table S4.** Treatment of *A. stephensi* larvae with 1, 10, and 100 µM ABA reduced lifespan of adult female mosquitoes relative to untreated controls, regardless of additional adult supplementation with 100 nM ABA. In accord with previous observations (Glennon et al., 2017), lifespan of adult females derived from untreated larvae and supplemented with 100 nM ABA in a weekly blood meal was not significantly different from females derived from untreated larvae and not supplemented as adults (Log-Rank p = 0.904). In the presence and absence of adult ABA supplementation, lifespan of females derived from larvae treated with 1 µM ABA was significantly reduced relative to adult females derived from untreated larvae (Log-Rank p = 0.006); similarly, in the absence of adult supplementation, lifespan of females derived from larvae treated with 1 µM ABA was significantly reduced relative to adult females derived from untreated larvae (Log-rank p = 0.028). However, there was no difference in lifespan of adult females derived from larvae treated with 1 µM ABA and supplemented or not as adults with 100 nM ABA (Log-Rank p = 0.958). In the presence of adult supplementation, lifespan of adult females derived from larvae treated with 10 µM ABA was reduced relative to females derived from untreated larvae (Log-Rank p = 0.005); similarly, in the absence of adult supplementation, lifespan of adult females derived from larvae treated with 10 µM ABA was reduced relative to females derived from untreated larvae (Log-Rank p = 0.025). However, there was no difference in lifespan of adult females derived from larvae treated with 10 µM ABA and supplemented or not as adults (Log-Rank p = 0.952). Finally, in the presence of adult supplementation, lifespan of adult females derived from larvae treated with 100 µM ABA was reduced relative to females derived from untreated larvae (Log-Rank p = 0.015); similarly, in the absence of adult supplementation, lifespan of adult females derived from larvae treated with 100 µM ABA was reduced relative to females derived from untreated larvae (Log-Rank p = 0.025). However, there was no difference in lifespan of adult females derived from larvae treated with 100 µM ABA and supplemented or not as adults (Log-Rank p = 0.116).
